# Genomic investigation of a suspected outbreak of *Legionella pneumophila* ST82 reveals undetected heterogeneity by the present gold-standard methods, Denmark, July to November 2014

**DOI:** 10.2807/1560-7917.ES.2017.22.25.30558

**Published:** 2017-06-22

**Authors:** Susanne Schjørring, Marc Stegger, Charlotte Kjelsø, Berit Lilje, Jette M Bangsborg, Randi F Petersen, Sophia David, Søren A Uldum, on behalf of the ESCMID Study Group for Legionella Infections (ESGLI)

**Affiliations:** 1 Department of Bacteria, Parasites and Fungi, Statens Serum Institut, Copenhagen, Denmark; 2 European Programme for Public Health Microbiology Training (EUPHEM), European Centre for Disease Prevention and Control, (ECDC), Stockholm, Sweden; 3 Department of Infectious Disease Epidemiology and prevention, Statens Serum Institut, Copenhagen, Denmark; 4 Department of Clinical Microbiology, Herlev Hospital, University of Copenhagen, Denmark; 5 Department of Virus and Microbiological Special Diagnostics; Statens Serum Institut, Copenhagen, Denmark; 6 Wellcome Trust Sanger Institute, Wellcome Genome Campus, Cambridge, United Kingdom

## Abstract

Between July and November 2014, 15 community-acquired cases of Legionnaires´ disease (LD), including four with *Legionella pneumophila* serogroup 1 sequence type (ST) 82, were diagnosed in Northern Zealand, Denmark. An outbreak was suspected. No ST82 isolates were found in environmental samples and no external source was established. Four putative-outbreak ST82 isolates were retrospectively subjected to whole genome sequencing (WGS) followed by phylogenetic analyses with epidemiologically unrelated ST82 sequences. The four putative-outbreak ST82 sequences fell into two clades, the two clades were separated by ca 1,700 single nt polymorphisms (SNP)s when recombination regions were included but only by 12 to 21 SNPs when these were removed. A single putative-outbreak ST82 isolate sequence segregated in the first clade. The other three clustered in the second clade, where all included sequences had < 5 SNP differences between them. Intriguingly, this clade also comprised epidemiologically unrelated isolate sequences from the UK and Denmark dating back as early as 2011. The study confirms that recombination plays a major role in *L. pneumophila* evolution. On the other hand, strains belonging to the same ST can have only few SNP differences despite being sampled over both large timespans and geographic distances. These are two important factors to consider in outbreak investigations.

## Introduction

Legionnaires’ disease (LD) is notifiable in Denmark. When *Legionella* isolates are obtained these can be voluntarily submitted to the Statens Serum Institut (SSI) for identification and typing. The surveillance system for LD combines information from the notifications with any respective available typing data. In the summer/autumn of 2014, Denmark observed an increase in LD cases compared with previous years [1]. In the North Zealand region, between July and November, 15 cases (75 cases/1.000.000/year) were notified. Comparatively, in the same period of the four previous years (2010 to 2013), an average of 5.5 (range 1 – 10) community-acquired LD cases were diagnosed (equivalent to 27.5 cases/1.000.000/year) in the region.

Among the 15 LD cases related to North Zealand in 2014, four were infected with *L. pneumophila* serogroup 1 subgroup Allentown/France, sequence type (ST) 82. ST82 has been only observed in six cases of LD since 2009 in different parts of Denmark but all outside the North Zealand region, and never in environmental samples. Three of the historical cases were associated with travel or were of unknown origin and three were community-acquired cases with no epidemiological links. Thus, the detection of four cases with ST82 within five weeks and an overall high incidence of LD in the region led to a hypothesis of a LD outbreak with a common environmental source.

In this study, we used standard epidemiological and typing tools as well as whole genome sequencing (WGS) to retrospectively investigate the putative LD outbreak.

## Methods

### Case definitions and data source

The European Union (EU) case definition for confirmed and probable cases of LD [2] was used. All cases in this study had pneumonia. Confirmed cases were diagnosed by culture and/or urinary antigen tests and probable cases were diagnosed by PCR only. The outbreak case definition was a person with LD diagnosed at the Department of Clinical Microbiology (DCM) of the regional hospital between 30 June and 19 November 2014 with no hospitalisation or travel during the incubation period (4 to 10 days before onset of symptoms). Information on confirmed/probable LD cases was extracted from the surveillance database at SSI and from the records of the DCM at the regional hospital.

### Epidemiological investigation

Basic information on travel and hospitalisation was collected for all patients. The four cases with culture-confirmed ST82 were interviewed using an extended questionnaire focusing on symptoms, risk factors, place of work, daily habits, traffic patterns and recent travel. Home addresses were obtained using the Danish Civil Register and confirmed during the interviews.

### Environmental investigation

At the beginning of January 2015, water samples were collected from the homes of three ST82 cases. One case had moved in the meantime and it was not possible to obtain water samples from the previous home. During the incubation period, one case, a professional cleaner, had attended work and water samples were also collected from this location. Two water samples each of 1 L were collected from each of the four addresses. Samples of hot water were collected from the shower hoses at the homes and tap water from the workplace of the cleaner as a first flush sample and an additional one after 20 s of flushing. The owners were instructed not to clean the shower before the visit or use it on the day of the visit. Additionally, a swab was taken from the tap of the shower. Samples were analysed at the National *Legionella* Reference Laboratory at SSI. During the visit, cases were re-interviewed, using an event calendar as a memory aid.

### Microbiological investigation

#### Culture of *Legionella* from water samples

Water samples were analysed in accordance with ISO 11731, which consisted of direct plating (2 x 0.5 mL) as well as plating (0.1 mL) concentrated (x100) sample material after filtration (0.2 µm filter), and (x1,000) after centrifugation. The material was seeded on selective media Modified Wadowsky Yee (MWY) and Glycine-Vancomycin-Polymyxin B sulphate-Cycloheximide (GVPC) agar plates (both from Oxoid, GmbH) and was incubated at 37 °C in a humid atmosphere for 7–8 days. If dense growth of background bacteria was observed, acid (HCl-KCl buffer, pH 2.2 for 5 min) and heat treatment (50 °C for 30 min) on the concentrated samples were applied.

#### Diagnostic methods at the Department of Clinical Microbiology

LD was diagnosed using a combinatory approach. First, real-time PCR was performed on respiratory samples to detect and differentiate between *Legionella* spp. and *L. pneumophila* [3]. PCR-positive samples were cultured for *Legionella* spp. on both Modified Wadowsky Yee-Oxoid (MWY-O) and Buffered Charcoal Yeast Extract (BCYE) agar plates (in-house media) by standard technique. Colonies identified as *L. pneumophila* with MALDI-TOF (MALDI-TOF) were referred to SSI for typing including serotyping by the Dresden panel (including MAb 3 of the international panel) of monoclonal antibodies to determine the serogroup and subgroup if applicable [4,5]. Urine samples were examined for *L. pneumophila* serogroup 1 soluble antigen (UAg) by the Alere BinaxNOW assay according to instructions from the manufacturer.

#### Sequence-based typing on clinical isolates and PCR-positive samples

Genomic DNA from the submitted isolates was extracted by the QIAamp DNA Mini kit (Qiagen) and *L. pneumophila* isolates were genotyped using the European Society of Clinical Microbiology and Infectious Diseases (ESCMID) Study Group for Legionella Infections (ESGLI) consensus sequence-based typing (SBT) scheme, which allows assignment of seven ordered alleles to an allelic profile representing a sequence types (ST) [6,7]. The trace files with the obtained sequences were analysed using the Legionella SBT quality tool at the website (http://www.hpa-bioinformatics.org.uk/legionella/legionella_sbt/php/sbt_homepage.php) to retrieve STs and ensure the quality of sequences. PCR-positive samples negative for L. pneumophila by culture were subjected to direct nested SBT as previously described [8].

#### *wzm* PCR (serogroup 1-specific PCR)

Available PCR-positive culture-negative samples were investigated by a real-time PCR targeting the serogroup 1 marker *wzm* [9,10] to discriminate between *L. pneumophila* serogroup 1 and non-serogroup 1. A positive result in the *wzm* PCR was considered as serogroup 1 whereas PCR-positive sample with a cycle threshold (CT) value of ≤ 35 in the specific *L. pneumophila* real-time PCR (*mip* specific PCR, in-house, SSI*)* but with no amplification with the *wzm* primers was considered as non-serogroup 1.

#### Whole genome sequencing

Genomic DNA used for SBT was also used for whole genome sequencing (WGS) using the Illumina MiSeq platform to obtain 251-bp paired-end reads according to the instructions from the manufacturer, or the Illumina HiSeq platform with 100-bp paired-end reads. The isolates of the four ST82 cases were initially analysed together with three other epidemiologically unrelated community-acquired ST82 isolates from Denmark; two cases from Funen in 2011 and one case from Jutland in 2012. None of the 488 genomes available at ftp.ncbi.nlm.nih.gov/genomes/refseq/bacteria/Legionella_pneumophila were ST82 and eligible for inclusion. However, additional sequences were subsequently included in the analysis - one sequence from a ST82 isolate from 2015, Jutland in Denmark, and four ST82 sequences from the United Kingdom (UK) [11], where one sequence originated from an isolate of a travel-associated case. This resulted in a total set of 12 whole genome sequences for the investigation (Table 1).

**Table 1:** Isolates that were analysed using whole genome sequencing (n = 12)

Case ID	Identifier^a^	Year	Country	Region	Acquired	Sample accession	Experiment accession
**Case 5**	EULV9728	2014	DK	Zealand	CA	ERR2009177	ERX2068934
**Case 6**	EULV9736	2014	DK	Zealand	CA	ERR2009176	ERX2068935
**Case 7**	EULV9737	2014	DK	Zealand	CA	ERR2009171	ERX2068936
**Case 11**	EULV9735	2014	DK	Zealand	CA	ERR2009170	ERX2068933
**DK_A**	EULV9728	2011	DK	Jutland	CA	ERR2009172	ERX2068937
**DK_B**	EULV10974	2011	DK	Funen	CA	ERR2009173	ERX2068938
**DK_C**	EULV10973	2012	DK	Funen	CA	ERR2009174	ERX2068939
**DK_D**	EULV10972	2015	DK	Jutland	CA	ERR2009175	ERX2068940
**UK_E ^b^**	EULV00167	2005	UK	London	UNK	NA	
**UK_F ^b^**	EULV3067	2008	UK	London	CA		
**UK_G ^b^**	EULV10052	2014	UK	South West	CA		
**UK_H ^b^**	EULV10407	2014	UK	UK	TA		
**Reference**	Lorraine NC_FQ958210^c^						

DK: Denmark; CA: community-acquired; United Kingdom: UK; UNK: Unknown; TA: travel-associated.

^a^ Sequence-based typing (SBT) data for all isolates were submitted to the European Society of Clinical Microbiology and Infectious Diseases Study Group for Legionella Infection (ESGLI)’s Database for *Legionella pneumophila* (http://www.hpa-bioinformatics.org.uk/legionella/legionella_sbt/php/sbt_homepage.php).

^b^ Sequence published in Mentasti et al. 2017 [11]

^c^ The GenBank accession number of the reference.

Identification of single nt polymorphism (SNP) variants was performed using NASP 1.0 (http://tgennorth.github.io/NASP/) by aligning sequence reads from the 12 *Legionella* isolates against the chromosome of *L. pneumophila* subsp. *pneumophila* str. Lorraine (GenBank accession number: NC_FQ958210) using the Burrows-Wheeler Aligner (BWA) [12] after removal of duplicated regions in the reference using NUCmer [13,14]. The Lorraine strain was chosen as reference as it was the closest closed reference available (as determined by k-mer analysis https://cge.cbs.dtu.dk/services/KmerFinder/); the Lorraine strain is ST47 and shares four of the seven SBT loci with ST82 (*flaA* (allele number 5), *asd* (number 22), *proA* (number 6) and *neuA* (number 6).

Variants were identified using the Genome Analysis Toolkit (GATK) Unified Genotyper, and all SNPs that did not meet a minimum coverage of 10 or that were present in < 90% of the base calls were excluded. High-density regions of SNPs including those derived from recombination events were removed using Genealogies Unbiased By recomBinations In Nt Sequences (Gubbins) v1.4.4 [15] with default settings. Phylogenetic trees were constructed using the maximum-likelihood algorithm implemented in PhyML at http://www.atgc-montpellier.fr/phyml-sms/with Smart Model Selection using the Bayesian Information Criterion with 100 bootstrap replicates. The Illumina sequences generated from the 8 Danish *L. pneumophila* isolates described in this study were submitted to the European Nt Archive (ENA; http://www.ebi.ac.uk/ena), accession numbers listed in Table 1 study ID PRJEB21315. Accessory genomes were analysed by using assembled Genomic reads by Spades v. 3.5.0 [16]. Prokka v. 1.2 [17] was used for gene annotation and Roary v. 3.6.0 [18] was applied to define gene ‘presence/absence’. Results were inspected manually.

## Results

### Microbiology and standard typing

Between 30 June and 19 November 2014, 15 patients fulfilled the outbreak case definition in North Zealand. According to the EU Legionnaires’ disease definitions, 13 of the cases were confirmed cases (6 by culture and seven by UAg test) and two were probable cases. Of the 15 cases, the isolates from four patients were typed as *L. pneumophila* serogroup 1, subgroup Allentown/France, ST82, as previously mentioned. These four cases were used to further define the cluster as a putative ST82 outbreak.

Based on subsequent typing data, five of the 15 cases could be excluded from the putative ST82 outbreak as the ST was not ST82. These included two culture-positive cases, one characterised as serogroup 3 ST87, and the other as serogroup 1, subgroup Benidorm, ST42. One culture-negative case was PCR-negative for *L. pneumophila* but positive for *Legionella* spp. (a non-*pneumophila* case). One culture-negative case was PCR-positive for *L. pneumophila* but negative in the *wzm* assay (and nested SBT revealed three of seven alleles confirming it as a non-ST82 case) and another was serogroup 1 (*wzm*-positive) but non-ST82 (nested SBT typing revealed five of seven alleles which suggested a ST42 [data not shown]).

Six cases were left as possible cases for the putative ST82 outbreak, all of which were diagnosed by urinary antigen (UAg) and two also by PCR (*L. pneumophila).* However, no sample material was stored for *wzm* PCR or nested SBT typing. In conclusion, the putative ST82 outbreak included six possible ST82 cases and four cases with culture-confirmed ST82 infection. In addition, no changes during 2010–2014 were identified in the laboratory procedure or diagnostic tests used for LD at DCM at the regional hospital which could have influenced the detection of LD cases.

### Descriptive epidemiology

**Figure 1:**
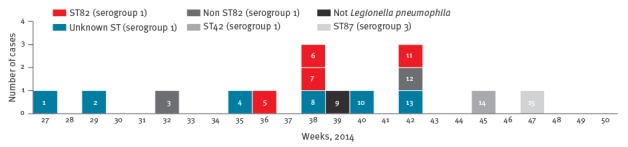
Cases with Legionnaires’ disease diagnosed at the Department of Clinical Microbiology, regional hospital, Denmark, 30 June–19 November 2014 (n = 15)

The mean age of the six possible (marked in dark blue in Figure 1) and four confirmed ST82 cases of the putative ST82 outbreak (marked in red in Figure 1) was 65.7 years, and eight of 10 cases were men. All patients were hospitalised. One of the 15 cases was fatal.

ST: sequence type.

Through the interviews, possible exposures during the incubation period were evaluated. No places of exposures related to shared events, local watermills, cooling towers, major constructions, or irrigation of recreational areas such as sports facilities etc., were in common to the cases, and therefore no additional environmental samples were collected.

**Figure 2:**
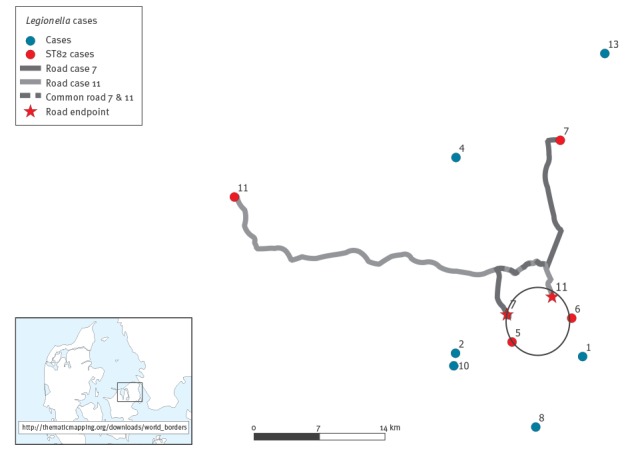
Mapping of residence of the cases of Legionnaires’ disease, or trajectory during their incubation period, Zealand, Denmark, 30 June–19 November 2014 (n = 10 cases)

The home addresses for all outbreak cases were plotted and major traffic patterns for the ST82 cases were included in the map (Figure 2). Two cases (number 11 and 7) had travelled approximatively 5 km on the same route by car during the incubation period. One case (number 6) mainly stayed in the town which case number 7 sometimes visited. However, there were no confirmed visits during the incubation period. In addition, case number 5 had remained close to home. Combined, this placed all four ST82 cases within a small geographical area of 7 km in diameter (‘the ST82 area’). The homes of the six possible cases were more distant, with a maximum of 42 km between case 13 and case 8. While case 8 had visited the southern part of the ‘ST82 area’ during the incubation period, in relation to work, the remaining five had not travelled to this area. However, cases 1, 2 and 10 lived close to the ‘ST82 area’, but the homes of cases 1 and 10 were separated by 13 km.

The circle on figure indicates a small geographical area of 7 km in diameter, which the four ST82 cases were linked to. Case 7 drove by car from point 7 to the location indicated by a red star shape (during the incubation period). Case 11 drove by car from point 11 to the location also indicated by a red star, leaving a common path of ca 5 km long between case 7 and 11.

### Environmental investigation

Analysis of the water samples revealed that one home was contaminated with L. pneumophila serogroup 1, subgroup Oxford/OLDA, ST1 (24,000 CFU/L in the first flush sample). The water samples from homes of the two additional cases were negative for *Legionella.* The water samples from the workplace were also contaminated with L. pneumophila serogroup 1, subgroup Oxford/ OLDA, ST1 but also with serogroup 4, subgroup Portland (> 600,000 CFU/L in the first flush sample). The workplace was notified of the finding, but no information of further sampling is available.

### Whole genome sequencing

**Figure 3:**
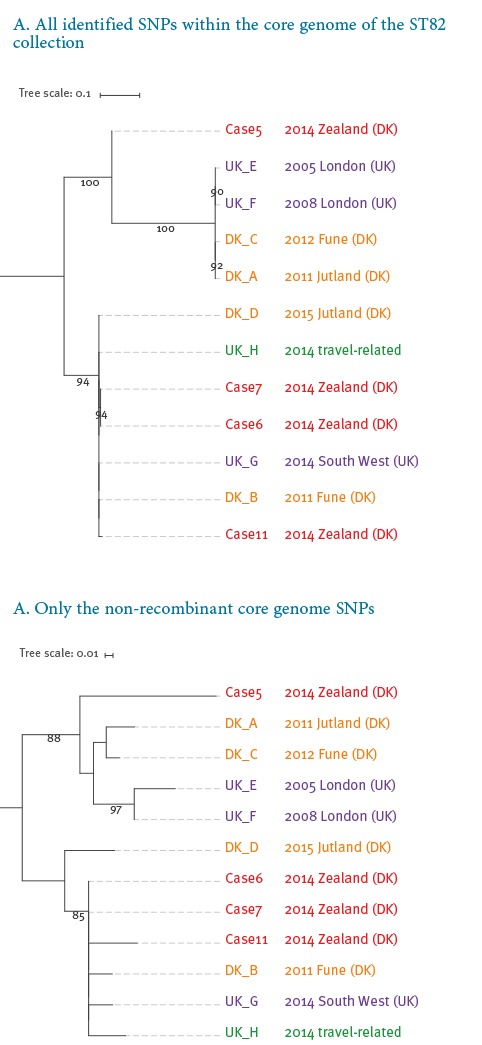
Phylogenetic analysis of the ST82 genomes demonstrates heterogeneity between the suspected outbreak isolates

SNP: single nt polymorphism; DK: Denmark; United Kingdom: UK.

The phylogenetic analysis (Figure 3), based on an alignment comprising > 90% of positions in the reference chromosome, showed that all included isolates could be separated into two main clades (I and II).

Clade I contains two isolates from Denmark obtained in years before the outbreak (DK_A, 2011 and C, 2012) together with two isolates from London, UK (UK_E, 2005 and F, 2008). Case 5’s isolate (clade I) is separated from the three other outbreak ST82 isolates located in clade II by ca 1,700 SNPs when putative recombinant regions are respectively included in the SNP analysis (Figure 3A). When such recombinant regions are excluded, this case’s isolate sequence differed from the others by 16 to 21 SNPs (Figure 3B).

Shown are rooted maximum likelihood phylogenies which were reconstructed using, A) all identified SNPs within the core genome of the ST82 collection, and B) using only the non-recombinant core genome SNPs. Names listed in red are the investigated outbreak strains of ST82. The scale bar indicates substitutions per site.

Clade II contains three of the ST82 outbreak isolates (case 6, case 7, and case 11) together with unrelated isolates from Denmark (DK_B, 2011 and D, 2012) and the South West region of the UK (UK_G, 2014), as well as an isolate obtained in the UK but associated with travel (UK_H, 2014). The isolates from cases 6 and 7 were identical, whereas case 11 differed by 118 and four SNPs (relative to isolates from cases 6 and 7) when recombinant regions were included and excluded, respectively. The two main clades (excluding case 5) were separated by ca 3,600 SNPs when recombination regions were included but only by 12 to 21 SNPs when these were removed. Thus, the genetic variation within the two clades due to de novo mutation was very limited.

**Figure 4:**
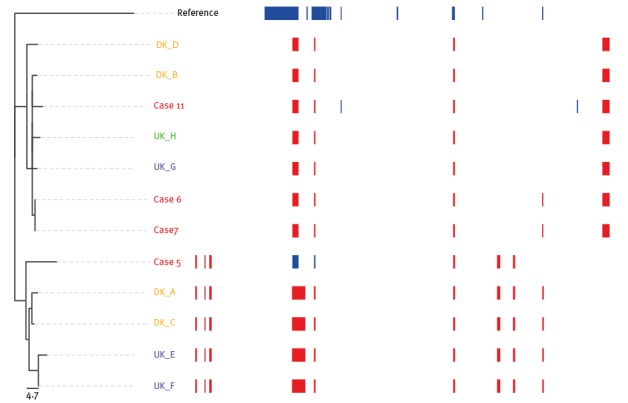
Predicted recombined regions in isolates of the ST82 lineage included in the investigation of a putative outbreak in 2014 conducted in Denmark

In clade I, all four strains shared the same putative regions of recombination (Figure 4); thus the intra-clade SNP distances were similar irrespective of whether these regions were included or excluded (3 to 9 SNPs). The isolate from case 5 shares recombinant regions with isolates in clade I (Figure 4), and clusters together with these isolates when recombinant regions are removed (Figure 3B); this is a strong indication of a common evolution for all the isolates in clade I and for the isolate from case 5.

DK: Denmark; United Kingdom; UK.

The reference is *Legionella pneumophila* subsp. *pneumophila* str. Lorraine, GenBank accession number NC_FQ958210.

A phylogenetic tree constructed using single nt polymorphisms (SNP)s that remain after the identification and removal of recombined regions by Gubbins algorithm is shown. The recombined regions are indicated by blue blocks, which are unique to a single isolate, and red blocks, which are shared by multiple isolates through common descent. The horizontal position of the blocks represents their position in the core genome alignment.

The observed variation within clade II was higher than within clade I with zero to 118 SNPs between isolates, compared with zero to 10 with or without recombinations, respectively. The isolates from outbreak cases 6, 7 and 11 display signals of recombination that were not shared by any of the remaining four isolates (DK_B, DK_D, UK_G and UK_H) in this clade (Figure 4). The identical isolates from cases 6 and 7 were phylogenetically more related to UK_G, UK_H and DK_B with only 43 to 44 SNP and two to three SNP differences with and excluding identified regions of recombination, than to the last suspected outbreak isolate of case 11 with 118 and four SNPs, respectively (Figure 3).

The maximum time span between the isolation dates of any two isolates in the same clade was four and a half years between DK_B and DK_D. No recombination events distinguishing these two isolates were identified in the analysis, and seven SNPs separated the isolates. In the analysis of the accessory genome, 540 genes were identified among the 12 isolates, however these contained no known pathogenic virulence genes. The analysis of the accessory gene content confirmed the relatedness between isolates from case 6 and 7 (data not shown).

## Discussion

In this study, we present the results of the epidemiological, environmental and genetic (typing) investigations of a small putative ST82 LD outbreak with four ST82 cases and six possible cases (one fatal) that occurred between June and November 2014 in Northern Zealand, Denmark.

Four cases of this suspected outbreak were caused by ST82.This ST is not common in Denmark and, as mentioned above, has not been seen in this geographical area before or after summer/autumn 2014. According to the ESGLI *L. pneumophila* SBT database, ST82 is not a very common ST in Europe or elsewhere with only 125 of 10,929 submitted isolates (as at 17 August 2016). All submissions are from Europe and mostly from France (n = 78) followed by UK (n = 12) and the Netherlands (n = 10). Only four of the isolates in the database are of environmental origin, however, none was obtained for this study. This distribution resembles that of the more common and closely related ST47 (which includes the Lorraine reference strain), with 612 entries of which only 10 are environmental. This type is the most common ST among clinical isolates in the Netherlands, England/Wales and Belgium [19] but many of the submissions are from France (n = 265).

The four ST82 cases in the putative outbreak clustered in regards to sero-/subgroup and ST (ST82), but, importantly, also in time and geographic region of residence and commuting. However, no common place(s) of exposure could be determined. We found *L. pneumophila* in water samples from sites where two patients had been during the incubation period, but none of the isolates were ST82. The lack of ST82 in the water samples (homes and workplace) could imply one or more external sources. The epidemiological data placed all four ST82 cases within a small geographical area of 7 km in diameter, thus the focus of the investigation was on these cases. Two of them had a common driving itinerary, along a route affected by road construction works which caused long queuing times, so it was speculated that cooling towers along their route might be the source of the outbreak. Both cooling towers and aerosolised water from industrial settings have previously been implicated in LD outbreaks [20–24]. However, in contrast to other European countries, Denmark does not have a cooling tower register, which limits the investigation and the environmental sampling from these sources [25]. No environmental ST82 isolates were obtained from any putative sources and no environmental ST82 isolates were available to be included in the analysis.

WGS was applied to obtain further clarification into the origin and relatedness of the four cases. It has recently been described that *L. pneumophila* can be rather genetically heterogeneous even among isolates within defined outbreaks [26,27]. However, a recent study of 10 separate *L. pneumophila* serogroup 1 ‘outbreaks’ in New York State [28] showed that isolates from almost all outbreaks formed outbreak specific clusters without any overlap, and isolates within these clusters differed by < 5 SNPs in most instances. In this investigation, a variety of isolates from unrelated cases of LD that occurred in both Denmark and the UK were included under the assumption that isolates from the same source would show less variation compared with epidemiologically unrelated isolates. However, our WGS-based phylogenetic analysis, performed before and after the removal of recombined regions, challenges the hypothesis of a common source for the four investigated ST82 outbreak isolates. The isolate from case 5 differs from the other three ST82 isolates by ca 1,700 SNP differences and at least six recombined regions. Secondly, cases 6 and 7 were identical based on our analyses, but more closely related to (i) a travel-associated isolate obtained from the UK, (ii) the UK isolate from 2014, and (iii) an epidemiologically-unrelated Danish isolate from 2011, than to the isolate from case 11. In addition, case 11 differs from cases 6 and 7 by three putative recombination events. Other *L. pneumophila* cases have been described as part of an outbreak based on strong epidemiological links with 15–17 SNPs between isolates [29,30].

As recombination has been shown to be a significant driving factor in *Legionella* evolution [31], it is important to consider this process when inferring relatedness based on core diversity. Our data indicate that the diversity within the ST82 clade is very limited when disregarding the effect of recombination and highlight the importance of including unrelated isolates of the same ST in the WGS analysis when investigating an outbreak in genetically highly similar clones. Around 99% of the SNP differences that distinguished the case 5 isolate from isolates from case 6, 7 and 11 were found in the recombinant regions. The most recent common ancestor of the isolate from case 5 and the isolates from 6, 7 and 11 must have existed before 2005 (9 years prior) as the oldest isolate in clade I is from 2005. Other publications also highlight the importance of evaluating and including observations of recombination in outbreak investigations [26,31]. McAdam and colleagues described one patient with two genetic subtypes which differed by 20 core genome SNPs (after removal of recombination events) during a cluster detection in Edinburgh and concluded, based on the short timescale between the exposure and isolation, that multiple subtypes must have co-existed in the source before acquisition [27]. Coscollá and colleagues also described mixed infections with different subtypes of *L. pneumophila* in outbreak patients [32]. Hence, the observed difference between the isolate from case 11 and two isolates from cases 6 and 7, respectively, is comparable to their findings and does not alone exclude a common source for the three cases. However, the results obtained by the inclusion of the epidemiologically unrelated isolates indicate that the cases could be unrelated (i.e. from different sources) despite their close genetic cluster.

Our finding that epidemiologically unrelated isolates sampled many years apart can differ by as few as two SNPs implies a very low evolutionary rate by point mutation for *L. pneumophila* (< 1 SNP/genome/year) and perhaps the existence of a dormancy stage within the life cycle. Underwood et al. also found that some isolates of the same ST (interestingly the close ST47) that were separated by several years and geographic location differed by just four SNPs [31]. The diversity could be different in other *L. pneumophila* lineages, however similar results have recently been shown in several other STs 1, 23, 36, 37, 47 and 62 [33,34].

WGS analysis has emerged as the new and highly discriminatory tool for microbial genotyping. Obtaining data, analysing and understanding the outputs can be challenging both in regards to timely analysis of data and due to the lack of standardisation, which makes rapid sharing of these cumbersome. Recent work, however, attempts to standardise the typing of *Legionella* [35]. The national surveillance in Denmark is still based on ST typing with subsequent WGS on selected clusters. Real time analysis by WGS may have ended the suspicion of a larger outbreak very early in the process as the isolate of case 5, the chronologically first ST82 case, was clearly different from the following two ST82 isolates, which on the other hand were indistinguishable. ST82 has not been detected in any cases in this region of Denmark either before or after this period in 2014. Therefore, the situation with four cases with the same rare ST diagnosed within a few weeks was extraordinary.

The six possible cases were not included in the WGS analysis, as the primary diagnoses were performed using UAg and no isolates were available. An attempt to culture the causative agent in all cases of LD is important, but even without isolation, the examination of positive respiratory samples is of great value to include or exclude cases from an outbreak as shown recently by Mentasti and co-workers [10] and in this study. Despite the fact that isolate submission is only voluntary in Denmark, the continued submission of isolates is of pronounced value for national surveillance, as well as submission of positive PCR samples where isolation is not possible.

We conclude that our data contribute to the discussion on how *L. pneumophila* outbreaks should be interpreted using WGS data and contribute to the general knowledge about the diversity within *L. pneumophila*. In this investigation, the microbiological results do not directly point to a single source outbreak and we were left without a clear epidemiological link. This highlights the importance of timely interviews of cases to explore all possible exposures. However, the presented data also show the importance of including epidemiologically and spatially unrelated isolates into WGS-based analysis as well detecting and evaluating the effect of recombination on the interpretation.
